# Facile One-Step Dynamic Hydrothermal Synthesis of Spinel LiMn_2_O_4_/Carbon Nanotubes Composite as Cathode Material for Lithium-Ion Batteries

**DOI:** 10.3390/ma12244123

**Published:** 2019-12-09

**Authors:** Chaoqi Shen, Hui Xu, Liu Liu, Heshan Hu, Siyuan Chen, Liwei Su, Lianbang Wang

**Affiliations:** State Key Laboratory Breeding Base of Green Chemistry-Synthesis Technology, College of Chemical Engineering, Zhejiang University of Technology, Hangzhou 310014, China; shenchaoqi@zjut.edu.cn (C.S.); 2111701106@zjut.edu.cn (H.X.); 2111801220@zjut.edu.cn (L.L.); zjuthuheshan@163.com (H.H.); yinianzhizhuo928@gmail.com (S.C.); suliwei@zjut.edu.cn (L.S.)

**Keywords:** spinel lithium manganese oxide, carbon nanotubes, dynamic hydrothermal, nanocomposite

## Abstract

Nano-sized spinel LiMn_2_O_4_/carbon nanotubes (LMO/CNTs) composite is facilely synthesized via a one-step dynamic hydrothermal approach. The characterizations and electrochemical measurements reveal that LiMn_2_O_4_ particles with narrow size distribution are well dispersed with CNTs in the composite. The LMO/CNTs nanocomposite with 5 wt % CNTs displays a high specific discharge capacity of 114 mAh g^−1^ at 1C rate, and the retention rate after 180 cycles at room temperature reaches 94.5% in the potential window of 3.3 to 4.3 V vs. Li/Li^+^. Furthermore, the electrochemical performance of the composite with 5 wt % CNTs at elevated temperature (55 °C) is also impressive, 90% discharging capacity could be maintained after 100 cycles at 1C. Such excellent electrochemical performance of the final product is attributed to the content of CNTs added in the hydrothermal process and small particle size inherited from pretreated MnO_2_ precursor.

## 1. Introduction

Li-ion batteries (LIBs) are extremely important components in our society as power sources for diverse portable electronic devices and electric vehicles [1,2,3]. The emerging market of energy storage systems in different fields requires LIBs with low cost, high rate capability, improved safety, and reliability [4,5,6]. Eftekhari has discussed the related resources, research and manufacture of lithium-ion batteries in detail [7]. Currently, Ni–Co–Mn (NCM) ternary oxides and LiFePO_4_ are two representative cathode materials extensively used in LIBs [8,9]. However, the risk of thermorunaway and high cost of NMC [10,11] and relatively low energy density of LiFePO_4_ [12,13] motivate people to design novel cathode materials. Although many researchers have devoted efforts to improve the capability of NCM, how to guarantee the supply of Co will become a critical issue in the future [7]. Considering the cheap price, high operation potential (4 V vs. Li/Li^+^), environmental friendliness and relatively high electrical conductivity, it is believed that the spinel LiMn_2_O_4_ (LMO) can act as the supplement of NMC and LiFePO_4_ [14,15,16], especially in the application scenarios requiring low cost but working with gentle operation intensity, e.g., the e-bike. 

Establishing a facile route to synthesize LMO cathode material with ideal capability is not easy. The main drawbacks of LMO are the structural instability induced by Jahn-Teller effect and dissolution of Mn^2+^, which result in poor cycling stability especially at elevated temperature (e.g., 55 °C) [17,18,19]. To solve such problems, nano-sized LMO are prepared to enhance the Li^+^ diffusion rate and improve structural stability, such as solvothermal [20], sol-gel [21], mechanical-chemical [22], and microwave sintering [23]. On the other hand, introducing modifications such as doping [24,25,26] and surface coating [27,28,29] is also quite universal. These measures are capable to optimize the crystal structure stability and suppress the dissolution of Mn^2+^ [30,31,32,33]. Recently, LMO/CNTs composites with excellent electrochemical performance have been reported [34,35,36]. Carbon nanotubes can not only increase the electronic conductivity of spinel LiMn_2_O_4_ but also improve the Li^+^ diffusion rate during charging and discharging [37,38]. With a solid-state synthesis route, carbon materials will be oxidized by oxygen or LiMn_2_O_4_ at high temperature, thus the hydrothermal process is the ideal method to obtain LiMn_2_O_4_/carbon composites in one step. However, a static hydrothermal method requires long reaction time (>48 h) and hard to obtain uniformly distributed LMO/CNTs composite with MnO_2_ precursor [39,40].

Herein, an improved dynamic hydrothermal process is developed by employing a one-step approach to synthesize nano-sized LiMn_2_O_4_/CNTs composites from MnO_2_ within 5 h. To a large extent, the morphologies of the final products are determined by MnO_2_ precursors. The small particle size of ball-milled MnO_2_ not only contributes to achieve faster hydrothermal reaction kinetics and minimize the impurities, but also realizes shorter lithium ion diffusion path during charging and discharging. CNTs are well-distributed in the nanocomposite as the stirring introduced into the reaction, and the amount of CNTs added into the hydrothermal system influences the electrochemical properties significantly. As a cathode material for LIBs, LiMn_2_O_4_/CNTs composite exhibits excellent cycling stability, even at elevated temperature 55 °C, which implies such material can be utilized as a candidate for energy storage applications. Thus the facile one-step dynamic hydrothermal synthesis method is an effective route to prepare composite materials with cheap solid precursors to apply not only in lithium-ion batteries but also other related fields.

## 2. Materials and Methods 

LiOH·H_2_O, MnAc_2_·4H_2_O, MnO_2_, and CNTs were purchased from Aladdin (Shanghai, China). The CNTs were pre-treated in an acid solution mixed with 18.4 M H_2_SO_4_ and 15.3 M HNO_3_ in a volume ratio of 3:1 at 50 °C for 3 h before use. Manganese oxide (MnO_2_, 3.9 g) was wet ball-milled with 5 mL ethanol in agate jar with a rotating speed of ca. 600 rpm for 3 h. The powder was collected as a precursor for a hydrothermal reaction after ethanol removed.

The 3.9 g ball-milled MnO_2_, 3.675 g MnAc_2_·4H_2_O, and 300 mL LiOH solution (0.2 M) were mixed in a 500 ml dynamic stainless steel autoclave then sealed. The autoclave was heated up to 200 °C for 5 h with temperature increasing at 2 °C min^−1^, and the stirring rate was set at 200 rpm. After hydrothermal treatment, LMO powder (noted as S1) was collected by centrifuging and washing with deionized water three times. This LMO sample (S1) was dried in the vacuum oven at 120 °C for 12 h. Two composite samples with CNTs (2 wt% for S2 and 5 wt% for S3, respectively) were obtained through the same procedure. As a comparison, pristine MnO_2_ without ball milling was also employed to synthesize spinel LMO (noted as S0), and the difference between two MnO_2_ precursors was investigated. 

The phase and crystallinity of the samples were identified by powder X-ray diffraction (XRD, X’Pert Pro, PANalytical, Almelo, Netherlands) with Cu Ka radiation (λ = 0.154056 nm) with 2θ angles from 10° to 80°. The morphologies of samples were investigated by scanning electron microscopy (SEM, Nova NanoSEM 450, FEI company, Hillsboro, OR, USA) and transmission electron microscopy (TEM, Tecnai G2F30 S-Twin operated at 300 kV, FEI company, Hillsboro, OR, USA). Thermogravimetry (TGA, SDT Q600 V8.2 Build 100, TA instruments, New Castle, DE, USA measurement was also conducted in airflow at a 10 °C min^−1^ heating rate to estimate the content of the CNTs in the composites.

The electrochemical properties of the LMO/CNTs composites were evaluated with CR2032-type coin-cells, which were assembled in an argon-filled glove box. LMO/CNTs, a lithium foil, and a porous membrane (Celgard 2400, Charlotte, NC, USA) were used as a working electrode, a counter electrode and a separator, respectively. The working electrodes of LMO/CNTs were fabricated by casting slurries of the LMO/CNTs powders (80 wt%), polyvinylidene fluoride (PVDF, 10 wt%) and Super P (10 wt%) mixed in N-methyl-2-pyrrolidone (NMP) onto a carbon-coated aluminum foil. They were then dried in a vacuum oven at 120 °C overnight. The electrolyte was composed of 1 M LiPF_6_ solution in ethylene carbonate (EC) and dimethyl carbonate (DMC) (1:1 by volume). The cyclic voltammetry (CV) and electrochemical impedance spectroscopy (EIS) were conducted utilizing an Iviumstat (Eindhoven, Netherlands) electrochemical workstation. The voltage range for CV measurement was from 3.5 to 4.5 V with a scan rate of 0.1 mV s^−1^. Furthermore, the coin cells were galvanostatically cycled using CT2001A (LAND Electronic Co., Wuhan, China) multi-channel battery test system in the voltage range from 3.3 to 4.3 V at room temperature and 55 °C. 

## 3. Results and Discussion

**Figure 1:**
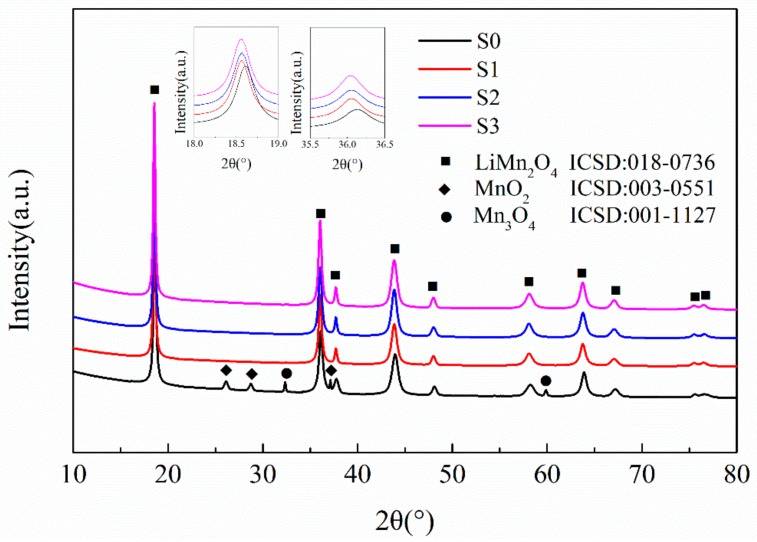
The X-ray powder diffraction patterns of the LiMn_2_O_4_/carbon nanotubes (LMO/CNTs) nanocomposites synthesized by the one-step hydrothermal process.

Figure 1 illustrates the XRD patterns of the LMO/CNTs samples synthesized via a one-step dynamic hydrothermal method. After the hydrothermal reaction, the obtained samples S1–S3 demonstrated diffraction peaks indexing to a well-crystalline spinel-type LiMn_2_O_4_ phase. The peaks of CNTs could not be detected, which indicates the amount of CNTs was too small to identify [40]. In comparison, XRD pattern of S0 via pristine MnO_2_ shows peaks of Mn_3_O_4_ and MnO_2_. This can be ascribed to the lithium ion in solution being harder to intercalate into the Mn-O matrix because of the bigger size scale and related slow solid–liquid reaction kinetics. Then the impurity Mn_3_O_4_ is formed, and the pristine MnO_2_ cannot be consumed completely [41]. Furthermore, the peaks around 18.5° and 36° in XRD pattern of S0 corresponding to (111) and (311) of spinel LiMn_2_O_4_ shifted to higher angles, which implies the lattice constant was smaller than the ones from ball milled MnO_2_. Such a result is attributed to the defects of Li ion generating more Mn^4+^ in the lattice of S0, and the ionic radius of Mn^4+^ is smaller than that of Mn^3+^ [42].

To understand the differences induced by two types of MnO_2_ precursors, XRD and SEM images of pristine and ball milled MnO_2_ were taken. The XRD patterns in Figure S1 show the pristine MnO_2_ powder with a pure ramsdellite phase (black line), and relatively broader peaks of ball-milled MnO_2_ indicates the size scale of MnO_2_ decreasing. The morphology of pristine MnO_2_ was irregular and the size scale ranged from 1 μm to 5 μm, while after ball milling, the particle size reduced significantly (Figure S2). Furthermore, SEM images in the same low magnitude of LMO final products related to these two precursors are shown in Figure S3, and which manifest that the S0 and S1 inherited size scale and morphologies from their corresponding MnO_2_ precursors for the secondary particles. Such results suggest the importance of MnO_2_ pretreatment [39,40]. High energy ball-milling enlarges the surface area of MnO_2_ to accelerate the solid–liquid reaction kinetics during the hydrothermal process, and the effect is assumed to produce less impurity and lead to better electrochemical performance.

**Figure 2:**
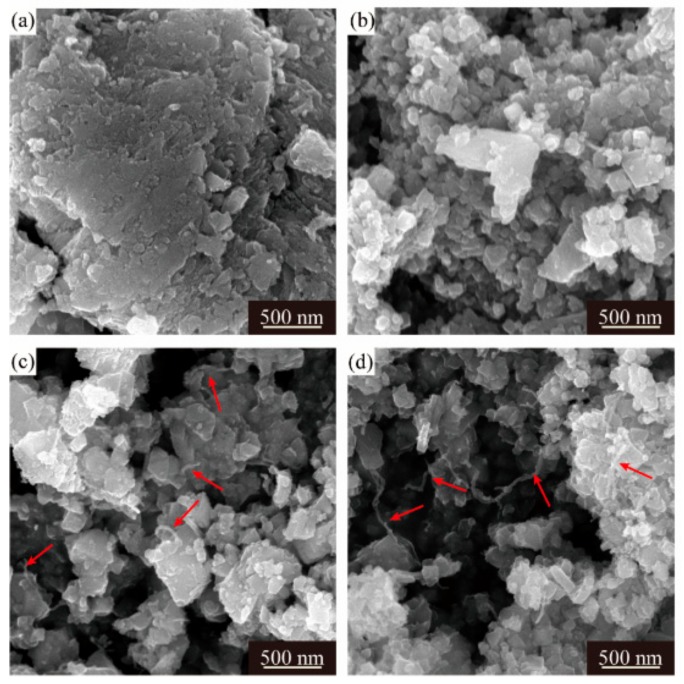
SEM images of four LMO/CNTs samples, (**a**–**d**) represent S0–S3, respectively.

**Figure 3:**
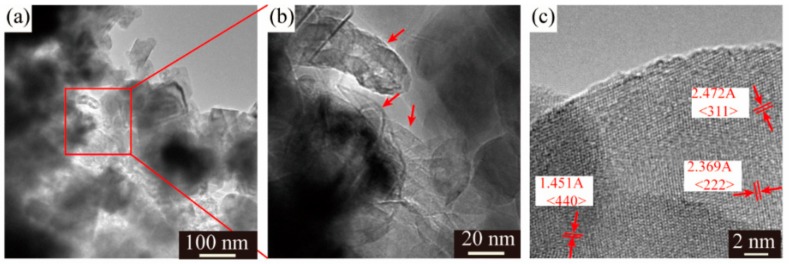
(**a**,**b**) TEM images and (**c**) HR-TEM of LMO/CNTs composite with 5 wt% CNTs.

Despite different morphologies of secondary particles, the state of aggregation of primary particles is also distinct from each other. The SEM images of the LMO/CNTs samples are presented in Figure 2. Sample S0 displayed large severely packed primary particles (Figure 2a), such a structure resulted in poor lithium ion diffusion path during electrochemical reaction. However, it is apparent that the primary particle size of S1 was distributed from 50 nm to 80 nm, although there were still some bigger particles involved (Figure 2b); the individual particles offered larger solid–liquid contact area to improve lithium ion diffusion rate. The morphology of multi-walled carbon nanotubes used is shown in Figure S4, the length of CNTs is several micrometers and the diameter is around 15 nm with 20 layers. LiMn_2_O_4_ crystals in S2 and S3 obtain similar morphologies as S1 (Figure 2c,d), while the carbon nanotubes in these two samples (red arrows in figures) are hard to distinguish as the diameter is too small [43,44] and most CNTs are embedded in the secondary particles from the core to the surface. The TEM images of S3 confirm the distribution of CNTs in composites (Figure 3a,b), carbon nanotubes interact with primary LMO particles firmly and establish a nice conductive framework. The high resolution HR-TEM in Figure 3c shows the lattice fringes corresponding to (311), (440) and (222) of spinel lithium manganese oxide.

**Figure 4:**
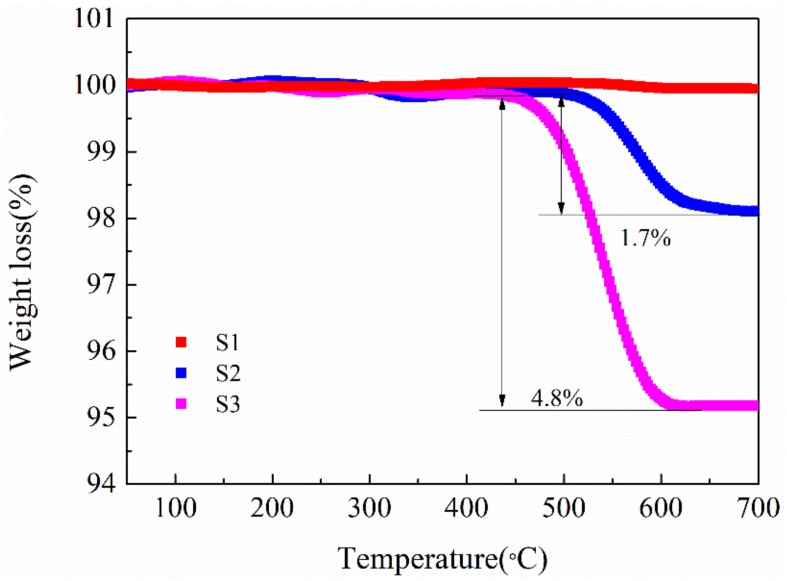
Thermogravimetry curves of the samples without (S1) and with CNTs (S2, S3).

To determine the content of CNTs in the LMO/CNTs composites, TG measurements were conducted with temperature ranging from 50 to 700 °C in airflow (Figure 4). S1 without CNTs does not show significant weight loss during the heating process, while for the composites S2 and S3, the measured values of CNTs content 1.7% and 4.8% are about 0.2%–0.3% less than those calculated from the starting compositions. The differences may be owed to material loss during the centrifuging step. As the carbon materials will be oxidized in air at high temperature, the content of carbon in LMO/C composite prepared under such condition is quite low and very hard to control [39,45]. We also find the CNTs reacting with LiMn_2_O_4_ in inert atmosphere, LiMnO_2_ and Mn_3_O_4_ appear in XRD pattern of S3 annealed at 600 °C for 4 h under Ar flow (Figure S5), which indicates LiMn_2_O_4_ is reduced by CNTs. Thus either in inert gas or with air, the LMO/carbon composite is hard to obtain with fine control via solid-state procedure, while hydrothermal route could facilely achieve given amount of CNTs in LMO/CNTs composite.

**Figure 5:**
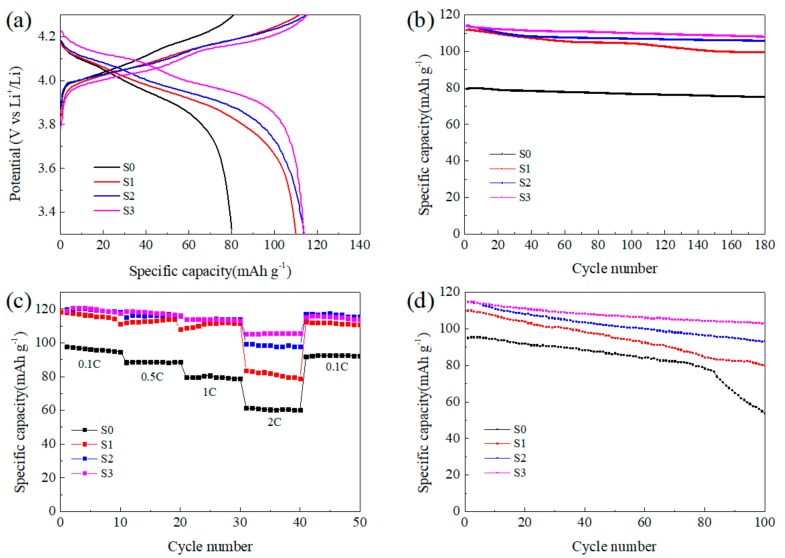
Electrochemical performance of the LMO/CNTs composites: (**a**) charging-discharging curves with 1C rate, (**b**) long cycling performance at 1C, (**c**) rate capability at room temperature and (**d**) long cycling performance at 1C rate under 55 °C.

The electrochemical properties of the synthesized LMO/CNTs composites are shown in Figure 5. There are two typical voltage plateaus of spinel LiMn_2_O_4_ in the charging-discharging curves shown in Figure 5a. During charging step, the lower plateau is associated to half of the Li^+^ ions extract from the tetrahedral sites in LiMn_2_O_4_ through Li–Li interaction to form Li_0.5_Mn_2_O_4_. Then, the rest half of lithium further extract without the aid of Li–Li interaction to generate λ-MnO_2_, thus the potential rises higher to form the second plateau [46]. The two plateaus during discharging in S0 are not clear due to the increasing polarization caused by lithium diffusion resistance [47], and it also demonstrates the limited high rate performance of this sample. The S0 from pristine MnO_2_ manifests poor specific capacity (80.1 mAh g^−1^ at 1C with efficiency of 98.99%), and the long cycling properties is unsatisfactory. This result reveals that the size scale of precursor influences the final product significantly, and the existence of impurity Mn_3_O_4_ in S0 also sacrifices the capacity as its inertness in the voltage window [48]. In comparison, S1 from ball-milled MnO_2_ exhibits a high reversible specific capacity of 111.8 mAh g^−1^ at 1C, even after 180 cycles the capacity maintained at 99.6 mAh g^−1^. Once CNTs were introduced into the reaction system, the electrochemical performance improved obviously. Although the specific discharge capacity at 1C was around 114 mAh g^−1^ for S2 and S3, after 180 cycles, the capacity retention rate maintained at 93% and 95% for S2 and S3 respectively, which was much better than S1 with the value of 89%. However, the energy efficiencies of S1, S2 and S3 were 98.56%, 98.86% and 98.65%, respectively, the effect of nano-sizing was not so obvious comparing to capacity. It is believed that the system kinetics improved with increasing active surface area to enhance specific capacity, while the ordered lattice in the bulk material favored the thermodynamics benefit to achieve high energy efficiency [49]. 

Figure 5c displays the rate performance of the final products, and the corresponding charging-discharging curves can be found in Figure S6. The S0 delivered the lowest capacity at all discharging rates, and LMO samples with small particle size achieved similar capacity at relatively low rates. While at 2C rate, S1 only showed capacity around 80 mAh g^−1^, the other two remain at 97 mAh g^−1^ and 104 mAh g^−1^, respectively. The charging–discharging curves in Figure S6 also illustrate the polarization during electrochemical reaction can be suppressed in LMO/CNTs composites and the existence of CNTs improved the rate capability effectively as enhancing the electronic conductivity of the active material [50]. The cells were cycled at 55 °C for 100 times with 1C rate to investigate the capacity retention performance under high temperature. As shown in Figure 5d, the retention rate was inferior to that at room temperature, which can be ascribed to the inevitable dissolution of Mn^2+^ into electrolyte being accelerated and degenerating the structural stability. The capacity of S0 decreased dramatically after 70 cycles in contrast, S1 maintained 73% after 100 cycles. This difference can be illustrated by the concentration of Mn^3+^ being suppressed with higher Li^+^ diffusion rate for S1, then particles with smaller size being more stable [51]. The existence of CNTs enhanced the thermal stability in a large scale, the capacity retention rates of S2 and S3 achieved 82% and 90% respectively, which implies the CNTs matrix could optimize the structural stability under high temperature. The improved electrochemical capability especially the cycling stability can be attributed to the small particle size and the addition of CNTs. The electron conductivity and lithium ion diffusion in bulk were optimized, then the concentration of Mn^3+^ could be balanced in the whole particle to suppress Jahn–Teller effect and relieve Mn^2+^ dissolution on the surface.

**Figure 6:**
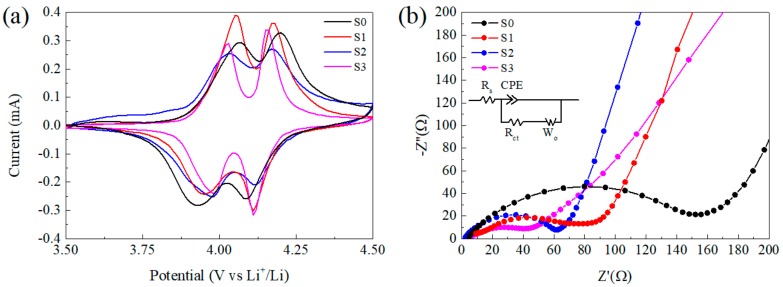
(**a**) Cyclic voltammograms (CV) of electrodes between 3.5 and 4.5 V vs. Li/Li^+^ at a scan rate of 0.1 mV s^−1^, (**b**) electrochemical impedance spectra of three half cells with different samples after the 5th cycle at 1C rate and the open-circuit voltage of the cells was 3.5 V.

Figure 6a displays the cyclic voltammograms (CV) of samples between 3.5 and 4.5 V vs. Li/Li^+^ at a scan rate of 0.1 mV s^−1^ after the 5th cycle at 1C rate. All of the CV curves show two redox peaks corresponding to the two-step of insertion and de-insertion of Li^+^ ions into and from the cubic spinel LiMn_2_O_4_ phase [52,53]. The peak shift corresponding to polarization of electrode was obvious for S0, which suggests low reversibility and fits well to poor cycling behavior. For S1–S3, it is interesting that the peaks around 4.1 V were close to each other, which implies the energy barrier of Li^+^ diffusion at this potential is analogous. However, the polarization around 3.95 V was distinct, S1 without CNTs showed the biggest potential gap of 0.12 V, while for S2 and S3, the gaps were 0.06 V and 0.04 V respectively. A similar result can also be found in the literature [47]. In conclusion, the addition of CNTs improved the lithium diffusion especially in the process of Li^+^ insertion and de-insertion between Li_0.5_Mn_2_O_4_ and LiMn_2_O_4_ phase, while for the next step with λ–MnO_2_ phase it was not so apparent and the mechanism requires further investigation. 

With the purpose to obtain a further understanding on the composite, electrochemical impedance spectroscopy (EIS) measurements were taken at a fully discharged state (Figure 6b). EIS patterns consist of a typical semicircle in high-frequency range corresponding to charge–transfer resistance and a straight line in the low-frequency range, which was related to the diffusion of Li-ions into the bulk of active mass [47]. The equivalent circuit is shown in the inset on Figure 6b and the electrochemical parameters for the alternating current EIS results calculated by the Z-view software are listed in Table S1. The value of R_s_ corresponding to the resistant of the cell is similar for all samples, while R_ct_ of S0 is the biggest (R_ct_ ≈ 139.5 Ω), which is only 70.4 Ω for S1. That means the decrease in particle size is able to accelerate the Li^+^ transfer, then lead to better electrochemical capability. Moreover, S2 and S3 with CNTs exhibit lower R_ct_ of 54.7 Ω and 43.5 Ω respectively, which can be ascribed to the enhanced charge transfer rate by the incorporated CNTs.

## 4. Conclusions

The LiMn_2_O_4_/CNTs (LMO/CNTs) composites are successfully synthesized using ball-milled MnO_2_ and MnAc_2_ as Mn precursors through a fast one-step dynamic hydrothermal process. Reducing the size scale of MnO_2_ precursor by ball milling and adding CNTs in dynamic hydrothermal procedure could improve the electrochemical performance of the final products effectively. As LiMn_2_O_4_ inherits the morphology of MnO_2_ precursor, the small particle size of MnO_2_ hinders the formation of Mn_3_O_4_ and MnO_2_ impurities due to the faster reaction kinetics, and the lithium diffusion path is shorter during electrochemical charging-discharging process. The introduction of carbon nanotubes in the LMO/CNTs composites can well confine the LMO nanoparticles from core to surface of secondary particles and construct electronic conductive network. The composite with 5 wt % CNTs shows the optimal electrochemical performance with discharge capacities of 114 and 106 mAh g^−1^ at 1C and 2C, respectively. Furthermore, the capacity retention rate under 55 ℃ could reach 90% after 100 cycles. Such a superior performance indicates that the LMO/CNTs composite has a great potential as a cathode material of lithium-ion batteries using in the fields with gentle operation intensity but requiring low cost, for example, the e-bike.

## Supplementary Materials

The following are available online at https://www.mdpi.com/1996-1944/12/24/4123/s1, Figure S1: the X-ray powder diffraction patterns of the MnO_2_ before and after ball milling, Figure S2: SEM images of the MnO_2_ (a) before and (b) after ball milling, Figure S3: SEM images of the LiMn_2_O_4_ synthesized via MnO_2_ (a) before and (b) after ball milling, Figure S4: TEM images of the multi-walled carbon nanotubes, Figure S5: the XRD patterns of S3 after annealing at 600 °C for 4 h under Ar flow, Figure S6: charge–discharge curves of different samples at various rate: (a–d) represent S0–S3 respectively, Table S1: Electrochemical parameters for the alternating current EIS results calculated using the Z-view software.
