# Supplementary material for: Facile One-Step Dynamic Hydrothermal Synthesis of Spinel LiMn2O4/Carbon Nanotubes Composite as Cathode Material for Lithium-Ion Batteries
Source: Materials (Basel). 2019 Dec 9;12(24):4123. doi: 10.3390/ma12244123 (PMC6947239; doi:10.3390/ma12244123)
Supplement: Supplementary file 1 [file materials-12-04123-s001.pdf]

materials

Supplementary Materials: Facile One-Step Dynamic Hydrothermal Synthesis of Spinel $LiMn_2O_4$/Carbon Nanotubes Composite as Cathode Material for Lithium-Ion Batteries

Chaoqi Shen, Hui Xu, Liu Liu, Heshan Hu, Siyuan Chen, Liwei Su, Lianbang Wang *

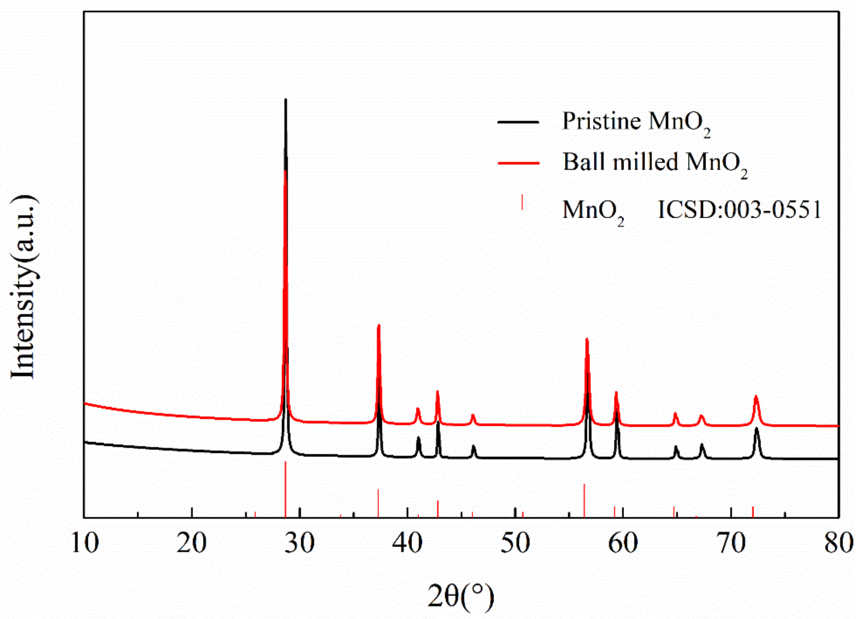

Figure S1. The X-ray powder diffraction patterns of the MnO_2 before and after ball milling.

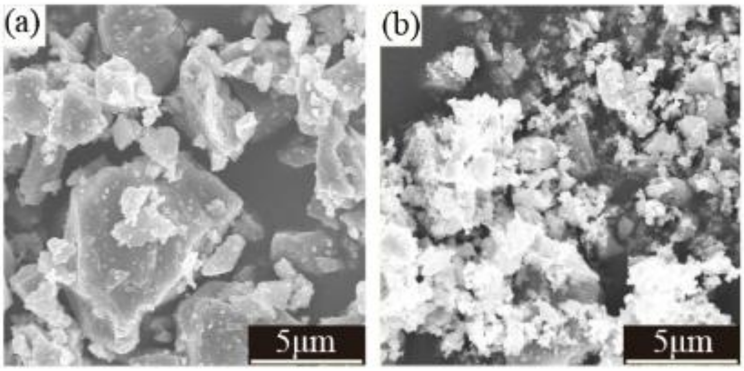

Figure S2. SEM images of the MnO_2 **(a)** before and **(b)** after ball milling.

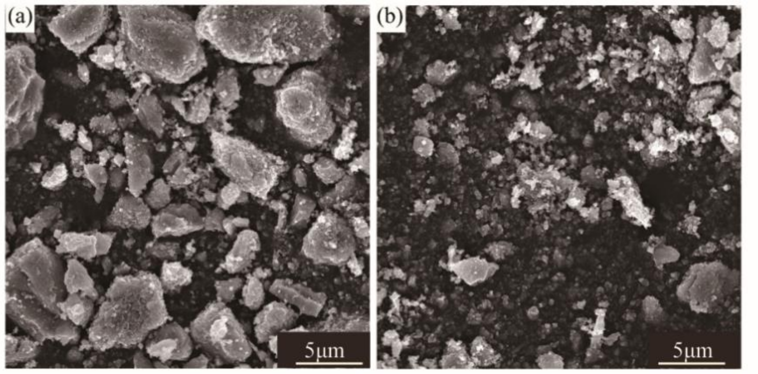

Figure S3. SEM images of the $LiMn_2O_4$ synthesized via MnO_2 **(a)** before and **(b)** after ball milling.

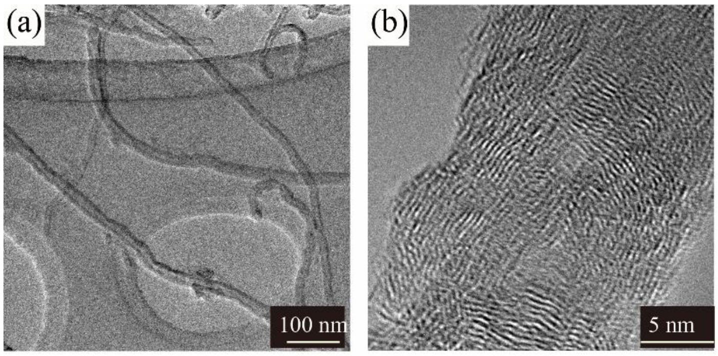

Figure S4. TEM images of the multi-walled carbon nanotubes.

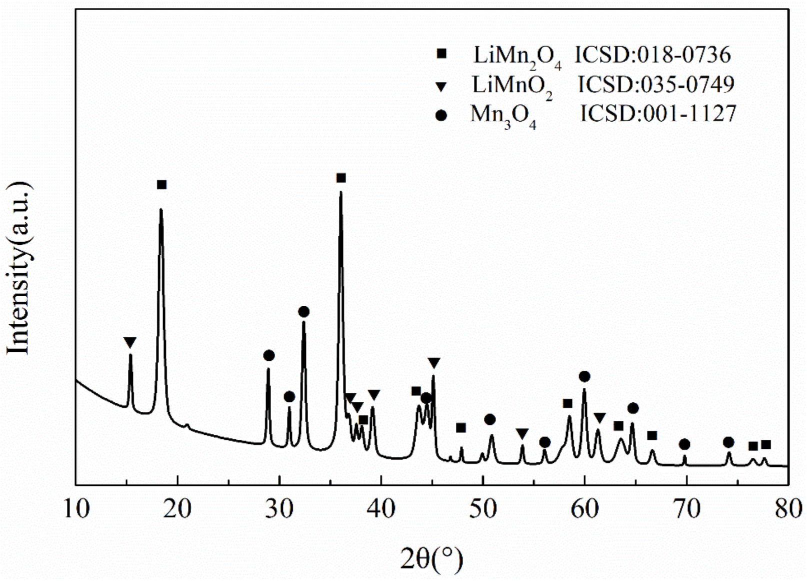

Figure S5. The XRD patterns of S3 after annealing at 600 °C for 4 h under Ar flow.

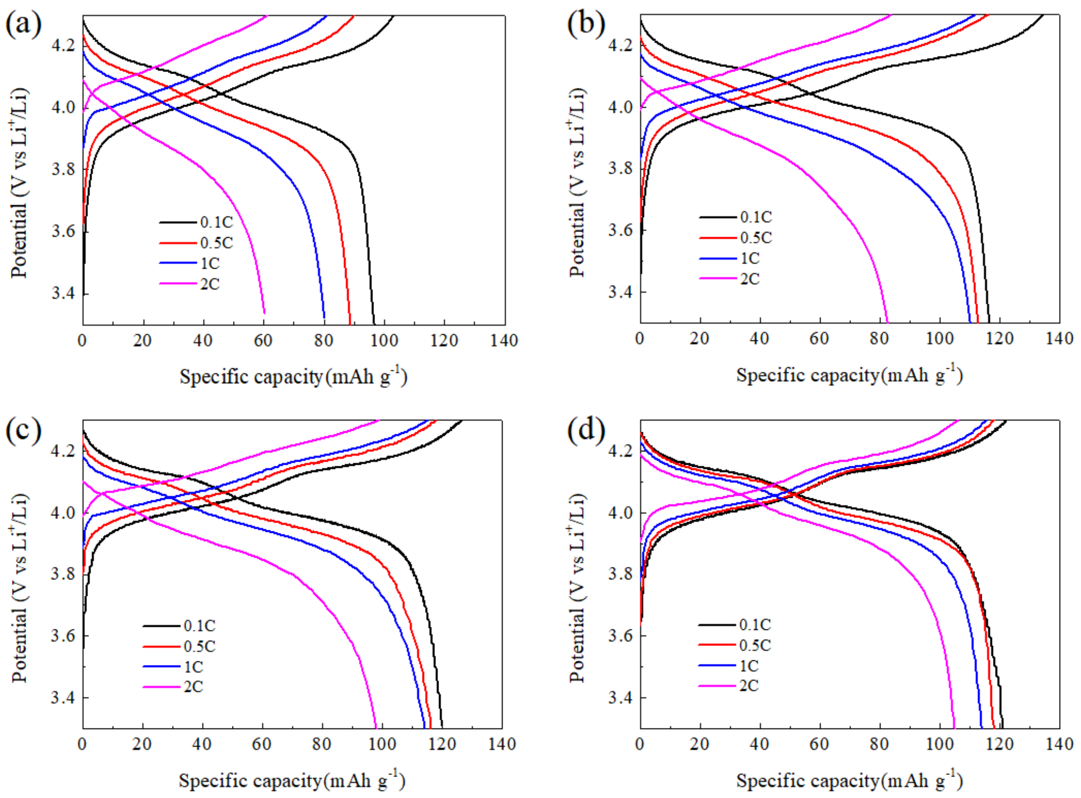

Figure S6. Charge–discharge curves of different samples at various rate: **(a–d)** represent S0–S3 respectively.

Table S1. Electrochemical parameters for the alternating current electrochemical impedance spectroscopy (EIS) results calculated using the Z-view software.

Samples	$R_s(\Omega)$	$R_{ct}(\Omega)$
**S0**	3.7	139.5
**S1**	5.3	70.4
**S2**	2.7	54.7
**S3**	4.2	43.5
